# BioSpring: An elastic network framework for interactive exploration of macromolecular mechanics

**DOI:** 10.1002/pro.70130

**Published:** 2025-04-18

**Authors:** Benoist Laurent, André Lanrezac, Hubert Santuz, Nicolas Ferey, Olivier Delalande, Marc Baaden

**Affiliations:** ^1^ Laboratoire de Biochimie Théorique Université Paris Cité, CNRS Paris France; ^2^ Laboratoire Interdisciplinaire des Sciences du Numérique CNRS, Université Paris‐Saclay Orsay France; ^3^ Institut de Génétique et Développement de Rennes (IGDR) CNRS, Université Rennes Rennes France

**Keywords:** biomolecular flexible docking, elastic network model, interactive molecular modeling, protein mechanics, protein–membrane interactions, real‐time simulation

## Abstract

BioSpring is an innovative tool for interactive molecular modeling and simulation, designed to explore the dynamics of biological structures in real time. Using an augmented elastic network model, the BioSpring framework enables researchers to intuitively examine complex biomolecules, and it combines real‐time feedback with the user's experience. This capability makes it ideal for initial analysis of molecular systems, protein–protein and protein–DNA docking, protein mechanics, and protein–membrane interactions. The multi‐resolution modeling approach combines accuracy and efficiency, supporting user‐driven analysis of molecular interactions, conformational flexibility, and structural mechanics. This framework improves upon traditional methods in terms of robustness, accessibility, and ease of use, while requiring only modest computational resources and enabling a fast turnaround time to obtain initial results. It provides insights into molecular function and dynamics that advance the field of structural biology. Source code, executables, and examples for the BioSpring simulation engine are available at https://biospring.mol3d.tech.

## 1 INTRODUCTION

Molecular simulation has become an essential complementary approach to experimental techniques to understand biomolecular systems. The spectrum of approaches ranges from very precise but computationally intensive methods to increasingly simple descriptions that can be calculated very quickly (Ingólfsson et al. 2014). Elastic network models (ENMs) represent an elegant balance between computational efficiency and physical accuracy in this spectrum of molecular simulation techniques (Bahar et al. 2010). Originally derived from normal mode analysis, ENMs have proven remarkably effective in reproducing the elastic behavior of proteins despite their conceptual simplicity (Tirion 1996). The fundamental insight underlying ENMs is that many functional motions of proteins can be described by modeling the biomolecule as a network of particles connected by springs, with each spring representing the molecular interactions that maintain the three‐dimensional structure. From a theoretical perspective, methods based on propagating atomistic coordinate representations operate on much shorter time scales and are computationally intensive. This limitation has led to the development and widespread use of lower resolution models such as ENMs. In the standard implementation of ENMs, the protein is reduced to a series of pseudo‐atoms (usually Cα atoms), with pairs below a certain cut‐off distance connected by Gaussian springs (Sanejouand 2011). Despite this drastic simplification, these models have provided valuable insights into protein mechanics and dynamics. The strength of ENMs lies in their scale independence—they can be applied equally well to all‐atom models, coarse‐grained representations, or simplified descriptions of protein structure using only alpha‐carbon. This flexibility allows researchers to choose the appropriate level of detail for a particular research question. In addition, ENMs have been shown to successfully capture large conformational changes and allosteric effects that are difficult to observe using more computationally intensive methods limited by shorter time scales (Fuglebakk et al. 2015).

BioSpring is a computational tool for interactive molecular modeling and simulation (Lanrezac et al. 2021) based on an augmented elastic network model (Tek et al. 2012), sometimes also referred to as the spring model. It was developed as a complement to computationally demanding conventional molecular simulations, offering an efficient real‐time method for interactively investigating large biomolecular structures or assemblies. The core concepts underlying the BioSpring framework were initially examined in an interactive system for identifying ion binding sites (Delalande et al. 2010). This system has shown the value of combining haptic feedback, where the user feels the forces exerted on different parts of the system thanks to a force feedback device, for example, and multi‐resolution molecular representations for intuitive analysis. The force feedback provided by haptic interfaces enhances the interactive exploration of molecular simulations by directly addressing the user's sensorimotor system and enabling intuitive manipulation of molecular structures through natural hand movements and tactile perception. The first designed spheric probe has later progressed to allow binding of molecular ligands. The elastic network model was chosen for its robustness (Leioatts et al. 2012) and computational efficiency, making it ideal for large‐scale applications. Building on this foundation, the BioSpring simulation engine now supports the study of biomechanical properties such as stiffness, flexibility, and allosteric effects. This capability plays an important role in the advancement of structural biology as it provides insights into protein interactions and functions at the molecular level. The latter are particularly valuable for integrative modeling approaches (Koukos and Bonvin 2019; Ziegler et al. 2021).

By integrating multiple levels of physical representation—such as electrostatic potential fields, volume densities, molecular accessibility at atomic and coarse‐grained resolution, and flexible probe models—the BioSpring framework allows users to focus on the key degrees of freedom within complex biological systems. This fine‐tunable multi‐resolution approach has already proven successful in a wide range of systems, from small proteins and pentameric ligand‐gated ion channels to large molecular assemblies such as viral capsids. The interactive nature of these BioSpring‐driven simulations, combined with an appropriate choice of physical representation, allows researchers to test hypotheses about molecular interactions, investigate conformational changes, and assess structure–function relationships. By enabling rapid user‐driven analysis, the BioSpring framework complements traditional automated methods and enables the rapid screening of protein–ligand interactions, biomolecular dynamics, and mechanical properties. The rigorous physical models underlying the BioSpring simulation engine ensure scientific validity, as demonstrated in previous studies of molecular recognition and binding mechanisms (Hemsath et al. 2005; Kozack et al. 1995; Tworowski et al. 2005; Wade et al. 1998).

User‐driven simulations complement conventional simulation techniques by enabling efficient investigation of conformational changes, mechanical properties, potential interaction modes, and detection of binding sites. With integrated visualization and optional haptic feedback, the BioSpring framework provides researchers with intuitive ways to rapidly investigate structure–function relationships and test mechanistic hypotheses. Such capabilities are particularly important in structural biology, where tools such as the BioSpring simulation engine can deepen our understanding of protein interactions at the molecular level in a rational way. The BioSpring framework emphasizes the importance of interactive, user‐friendly computational tools that use simplified molecular representations to facilitate real‐time investigation and analysis and make complex protein research more accessible.

The main purpose of this manuscript is to present BioSpring as a mature, robust, and reliable molecular simulation engine that is now ready for widespread adoption by the research community. Although the BioSpring software has a long development history dating back to before 2010, it has evolved into a stable, well‐tested tool. As a secondary goal, we emphasize the broader utility of augmented elastic networks as a theoretical framework for interactive molecular simulations and show how the implementation of this approach enables efficient and accurate molecular modeling.

## 2 MATERIALS AND METHODS

This section introduces the main methods and components of both the BioSpring framework and simulation engine. We begin with a very brief introduction to elastic networks, followed by a description of BioSpring's molecular simulation engine, which uses an advanced elastic network model to computationally efficiently capture the dynamics of complex systems. We then explain the key features to facilitate interactive simulations, followed by a description of the typical setup for running the BioSpring simulation engine, including the integration of visualization, haptic feedback, and computational modules. We describe how users can interact with models during simulations by using various input devices to directly manipulate molecular structures. Finally, we summarize the resources available to support BioSpring users, including documentation, sample environments, and interactive chatbot help for real‐time troubleshooting.

### 2.1 Elastic network models

Elastic network models are a simplified representation in which the molecular structure is maintained by interactions that are mimicked by springs. In the simplest model, each amino acid is represented by a single site, and a spring is placed between two sites if the distance between them is less than a certain value. It should be noted that this model does not take into account the connectivity of residues within the protein. Moreover, in the initial structure all springs are relaxed, which means that the system is in equilibrium. The Hookean pairwise potentials are set up between selected neighboring particles in such a way that the further the distance $d_{i,j}$ between two particles *i* and *j* deviates from their equilibrium distance $d_{i,j}^{0}$, the higher the associated potential energy $V\left(d_{i,j}\right)$ is,

(1) $$V\left(d_{i,j}\right)=\sum_{i<j}K{\left(d_{i,j}-d_{i,j}^{0}\right)}^{2}.$$

As mentioned above, the equilibrium distance $d_{i,j}^{0}$ is defined as the initial distance between particles *i* and *j*. By definition, this force tends to return the system to its equilibrium state, the initial conformation of the molecule. The sites used for an elastic network are generally the alpha carbons for proteins, and the value for the limiting distance to account for a spring typically varies between 7 and 15 Å. A single and identical force constant *K* is usually used for all pairs. In this representation, the number of degrees of freedom in the system is greatly reduced compared to a typical molecular dynamics simulation setup, for example.

### 2.2 Description of the BioSpring molecular simulation engine

The BioSpring simulation engine uses a spring network model, known as elastic network model (Chennubhotla et al. 2005), to simulate the simplified dynamics of interconnected beads. Traditionally, these beads are just mass‐ and size‐less interconnecting nodes. Springs act as a simple harmonic potential connecting closely located beads. This approach offers relative flexibility at the macromolecular level while preserving the overall shape of the system, significantly reducing the computational cost of simulations. The underlying theory and the equations implemented in the software are discussed in detail elsewhere (Lanrezac and Baaden 2023; Molza et al. 2014; Tek et al. 2012). In BioSpring, however, we expand this concept further, as briefly described in the following paragraph, giving a compact overview.

**FIGURE 1:**
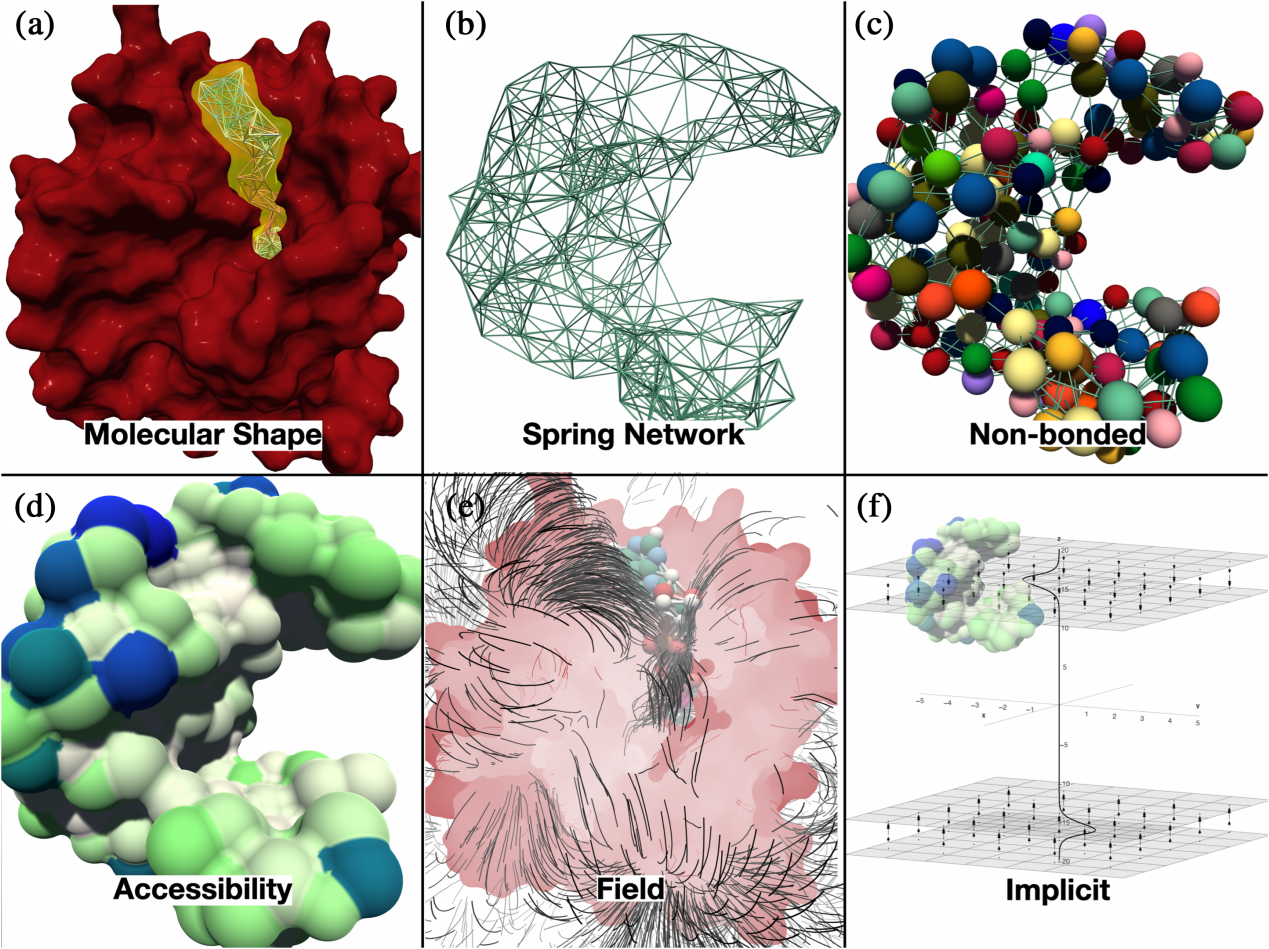
Key simulation features in the BioSpring framework, illustrated using the example of the enzyme guanylate kinase (GK). Panel (a) shows molecular shapes where an elastic network of the GK ligand (yellow area) is constrained by the static spatial constraints of the enzyme. Panel (b) shows an elastic spring network for the GK enzyme that implements flexibility of the model. In panel (c), non‐bonded terms are used to augment the nodes of the elastic network. They allow the observation of metastable states and intermolecular interactions that go beyond the simple energy landscape of a purely elastic network model, which always restores the initial state. Panel (d) shows the GK surface, colored according to the accessibility of the solvent. This property can be computed dynamically and used as a penalty to drive the system to match complementary hydrophobic/hydrophilic surface elements. In panel (e), field lines are shown to visualize electrostatic or gravitational‐like terms that can act on the non‐bonded beads, here the ligand of GK, to drive them to low‐energy regions. Panel (d) shows an implicit term that defines membrane‐related forces. It can control the positioning of the molecules, as in the GK enzyme shown in the diagram. Image under CC‐BY license by Marc Baaden.

In this approach, illustrated in Figure 1 (see “Spring Network”), a network of springs is constructed between reference particles in a molecular system, which are often alpha carbons for proteins and phosphorus atoms for oligonucleotides, using experimental structural data, typically from the Protein Data Bank (PDB) (Berman et al. 2000; Berman et al. 2003). The springs are generated between particles within a certain distance (cutoff distance), generally between 7 and 15 Å (Jeong et al. 2006), and multiple layers of springs can be combined (Delalande et al. 2018; Dubanevics and McLeish 2022). The initial equilibrium spring length and stiffness are determined based on experimental data or adjusted to achieve the desired system behavior and to stay within the relevant biophysical energy range for bonded interactions (Dubanevics and McLeish 2022; Li et al. 2016; Yang et al. 2006). At each time step of the simulation, forces are calculated for all particles in a given dynamic set, within a possible static set of immobile particles (depicted as “Molecular shape” in Figure 1). These forces include spring forces between connected particles. The BioSpring framework provided one of the first approaches to augment such a spring network by combining the elastic forces with extended multiscale interaction terms (illustrated as “Non‐bonded” in Figure 1) back in 2010 (Delalande et al. 2010). The latest version includes Van der Waals interactions, Coulomb forces between charged particles, membrane‐anchoring terms for implicitly represented arbitrarily shaped bilayers (“Implicit” in Figure 1), forces resulting from precomputed potential fields (e.g., electrostatic Poisson‐Boltzmann potentials, see “Field” in Figure 1), density force field for fitting a target to envelopes coming from cryo‐electron microscopy or small‐angle X‐ray scattering (SAXS) experiments, aggregation terms based on surface accessibility (“Accessibility” in Figure 1) and arbitrary user‐defined external forces for interactive manipulation. Therefore, we emphasize the originality of the model, which goes beyond simple point‐particle representations by considering not only steric and electrostatic interactions through explicit consideration of non‐bonded van der Waals and Coulomb forces but a broader range of features at multiple scales.

### 2.3 Features supporting interactive simulations

The BioSpring simulation engine's interactive capability is achieved through optimized computational strategies that enable fluid, real‐time coordinate updates. These optimizations include the offline calculation of potential maps and the implementation of a 3D grid system for efficient particle neighborhood calculations to limit the time for updating an index of nearby particles at each time step. The software is parallelized using OpenMP (Chapman 2014). The framework primarily uses coarse‐grained elastic network representations in which macromolecules, which can consist of several thousand atoms, are simplified so that multiple atoms are merged into a single bead or “grain.” Those grains are then linked together using harmonic potentials, that is, “springs,” as described earlier. This model provides an effective balance between computational efficiency and physical accuracy for interactive simulations, as shown previously (Delalande et al. 2009). Although all‐atom representations are supported for certain applications, the coarse‐grained approach is particularly suitable for real‐time manipulation and analysis. Mixed resolution approaches can be implemented to maintain atomic precision locally, at a site of interest, and simplified representations more remotely. User interaction can be further enhanced by haptic force feedback mechanisms or immersive environments, such as virtual reality interfaces, which allow direct manipulation of molecular structures during simulation. Such haptic feedback during manipulation provides the user with immediate tactile information about structural resistances and conformational flexibility, which naturally steers the user into energetically favorable paths when making structural changes.

### 2.4 Setting up an interactive experiment

**FIGURE 2:**
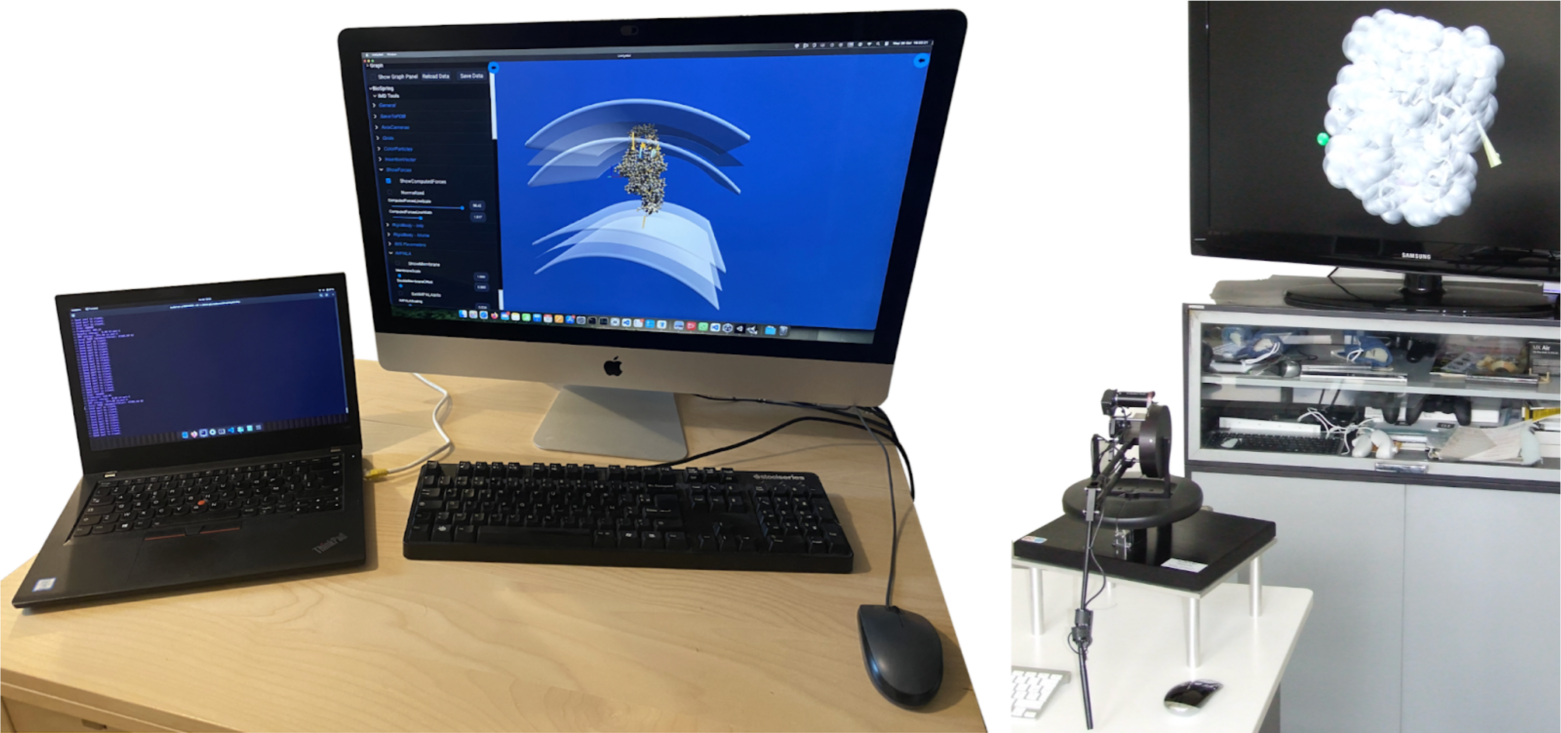
Alternative configurations for BioSpring simulation engine and visualization. (left) A distributed workflow where BioSpring simulations running on a laptop stream data in real time to a separate desktop workstation for visualization. (right) An advanced interactive setup combining haptic force‐feedback controls for precise molecular manipulation with stereoscopic visualization on a 3D display, enabling immersive molecular experiments with tactile feedback. Image under CC‐BY License by André Lanrezac, Nicolas Férey, and Marc Baaden.

The BioSpring simulation engine is designed to run on standard workstations, taking advantage of parallel processing capabilities where available. A typical setup integrates visualization, optional haptic feedback, and computational components on a single machine, with each component provided by a separate software application. Typically, software components would comprise UnityMol (Baaden 2024) for visualization, VRPN (Taylor et al. 2001) (integrated with UnityMol) as a client for compatible haptic devices, and BioSpring for computation. For optimal performance, a dedicated accelerated graphics card is recommended for the visualization component. All software components are connected over the network to a middleware such as MDDriver (Delalande et al. 2009) and can be run either on separate machines or on a single computer. Thanks to the MDDriver API, it is possible to adjust certain simulation parameters on the fly, such as viscosity, interaction forces, or the curvature of an implicit double membrane. At the same time, calculated data can be received from the engine, including energies, accessible surfaces, or particle insertion depth, enabling real‐time analysis. The hardware requirements of the system are modest, making it suitable for typical desktop and mobile environments. Two typical setups are shown in Figure 2. We provide ready‐to‐use environments of all software components through MolPlay (Baaden 2024), a dedicated bootable turnkey platform, along with tutorials, step‐by‐step guides, and walkthrough videos.

### 2.5 Interacting with the molecular system during a simulation

An interactive experiment allows the user to interact with molecular simulations in real‐time through various input methods. The simplest approach uses mouse input to apply force constraints to guide particles along two screen dimensions. For advanced three‐dimensional control, spatial input devices can be used to select and manipulate particles directly in 3D space (Delalande et al. 2009). Optionally, haptic devices can provide force feedback, although this is not required for the core functionality of the software. The interaction model follows established approaches used in molecular visualization software such as VMD (Humphrey et al. 1996). It allows the user to apply forces to individual particles or selected groups. When particles are selected, the simulation applies forces proportional to the distance between the geometric center of the selected particle group and the position of the input device. This implementation is based on an elastic model where the external forces are calculated and sent to the BioSpring simulation engine. This interaction approach maintains simulation stability regardless of the input method or frame rate, as the force calculations are decoupled from the update frequency of the visualization. This design ensures smooth user interaction while maintaining physical accuracy in the molecular simulation.

### 2.6 Mechanisms to support users

User support for both the BioSpring simulation engine and the BioSpring framework is provided through various channels. Traditional documentation includes scientific publications describing use cases or methods, focusing on the framework aspect (e.g., Delalande et al. 2018; Lanrezac and Baaden 2023; Molza et al. 2022; Saladin et al. 2010) as well as dedicated websites for the BioSpring simulation engine (https://biospring.mol3d.tech) or the MolPlay environment (https://molplay.mol3d.tech) that encompass the simulation engine but also interaction and visualization components needed for interactive experiments as well as several ready‐to‐use examples. The source code of the BioSpring simulation engine is available through Github (https://github.com/LBT-CNRS/biospring). User support and bug reporting are handled via GitHub's integrated tools, in particular the issue tracker for bug reports, feature requests, and general technical assistance. In addition, we have implemented an experimental chatbot on the BioSpring website that utilizes the Chatbaby framework to provide instant, interactive support. This trainable chatbot helps users navigate the features of the BioSpring simulation engine and of MolPlay and can provide some extent of real‐time assistance with setup and troubleshooting technical issues. The chatbot complements existing documentation by providing immediate, context‐specific support as users work with the tools.

## 3 RESULTS

The BioSpring framework enables interactive molecular modeling across multiple scales and application domains. In this section, we first introduce the basic architecture that allows BioSpring to represent different macromolecular structures with varying levels of detail and flexibility. We then demonstrate the versatility of the framework through three representative use cases that highlight different aspects of interactive molecular modeling: exploring protein dynamics, assembling protein–DNA complexes, and inserting membrane proteins into bilayers. These use cases are provided as ready‐to‐use examples with the software distribution and have been tested on various computer platforms. Detailed video walkthroughs are provided on the MolPlay website https://molplay.mol3d.tech. Many more experiments have been performed on various problems and systems that can be analyzed using this approach. These will be included as illustrated examples in the BioSpring software distribution. Briefly, BioSpring has proven to be versatile in various molecular systems and enables the localization of ion binding sites, the analysis of mechanical properties, and the investigation of allosteric effects. The tool supports flexible docking of small biomolecules, such as sugars or metabolites, and facilitates the analysis of protein–ligand interactions. It handles complex tasks such as semi‐flexible protein/DNA and protein/protein docking and has been used to simulate the deformation of viral capsids, mirroring atomic force microscopy (AFM) indentation experiments. For integrative modeling, the BioSpring simulation engine incorporates experimental data from SAXS and cryo‐EM to support flexible fitting of dynamic complexes such as filamentous actin and its protein partners.

### 3.1 Flexible multi‐scale framework for macromolecular design

The BioSpring framework implements a modular architecture that combines different levels of structural representation with customizable force and interaction fields. At its core, the framework uses a network of particles connected by springs, which can represent molecular structures at different scales—from all‐atom to coarse‐grained models. This elastic network approach is complemented by additional interaction terms that capture specific molecular properties such as electrostatics, excluded volume effects, and interactions with the environment. The framework allows seamless integration of these different components, allowing the user to choose the most appropriate combination of representations and forces for their specific application. Table 1 provides a schematic overview of these features by grouping them according to particle‐based, field‐based, and implicit terms contributing to the propagation of particles and molecules during a simulation with the BioSpring simulation engine that implements the framework's features.

**TABLE 1:** Overview of features for creating user‐defined multiscale molecular representations (see Figure 1 as well).

Feature	Level	Scale	Interactions	Description	Experiments	Use case examples
Particle‐based representations						
Molecular shape constraint	Beads	0.1–10 nm	Van der Waals, Coulomb, Springs	Fixed molecule shapes can be represented as beads at all‐atom or various coarse‐grained scales	FRET, NMR	MutS (Baaden 2022)
Springs	Beads	1–20 nm	Springs	Combine multiple levels of elastic spring networks interconnecting beads	FRET, NMR	Dystrophin (Delalande et al. 2018; Mias‐Lucquin et al. 2020; Molza et al. 2014), RecA/DinB (Saladin et al. 2010; Tashjian et al. 2019)
Non‐bonded	Beads	0.1–10 nm	Van der Waals, Coulomb	Spring network augmented by beads with non‐bonded interactions	FRET, NMR, molecular footprint, mutagenesis, chemical cross‐link	Dystrophin (Delalande et al. 2018; Mias‐Lucquin et al. 2020; Molza et al. 2014) and MC1 (Paquet et al. 2014)
Rigid bodies	Beads	1–100 nm	Van der Waals, Coulomb	Use a static bead‐based rigid body	FRET, NMR	OmpA (Lanrezac et al. 2022; Lanrezac and Baaden 2023) and Dystrophin (Delalande et al. 2018; Mias‐Lucquin et al. 2020; Molza et al. 2014)
SASA	Beads	0.1‐0.5 nm	Pseudo‐hydrophobic (experimental)	Compute static or dynamic solvent‐accessible surface area of beads	Cryo‐EM	Maltoporin (Lanrezac et al. 2022; Lanrezac and Baaden 2023)
Field‐based approaches						
Fields	Grid	1–100 nm	Electrostatic or gravitational potential grid	Beads “feel” forces based on field type and non‐bonded properties	Cryo‐EM, SAXS	DNAse (Delalande et al. 2010), Ryanodine receptor (Molza et al. 2022), and Dystrophin (Delalande et al. 2018)
Implicit representations						
Membrane	Implicit	10–1000 nm	Analytical function for membrane forces (example: UNILIPID method)	Arbitrarily shaped (multiple) membranes can be implicitly represented	Cryo‐EM	MetY (Lanrezac and Baaden 2023) and Neurotensin (Da Costa et al. 2013)

*Note*: The table organizes the features into three categories—particle‐based, field‐based, and implicit representations. For each feature, the table specifies the level of representation, the approximate applicable length scales, the relevant interaction terms for energy and force calculations, and a brief description of the feature. In addition, the types of experimental data that can be integrated with each feature are given along with practical use cases for some of them and related scientific references. Table under CC‐BY license by Marc Baaden.

The user can combine appropriate terms in a given calculation, and additional features may be added in future versions of the BioSpring simulation engine code along the lines of the classification discussed in an earlier report on multilevel modeling of biological systems (Baaden and Lavery 2007). We focus on the levels of representation that currently provide a flexible framework for experimentation. Table 2 describes examples of feature combinations that have proven useful in our research to date. Alternatively, it is possible to extend the range of methods for generating conformations. For specific applications, we have implemented systematic search and Monte Carlo‐like approaches which enable efficient automated sampling through a coarser rigid‐body based scheme, with faster coverage of the conformational space. We used such non‐interactive approaches for methods development and validation.

**TABLE 2:** Applications, their feature combinations and brief description.

Application example	Shape	Spring	Non‐bonded	Rigid body	SASA	Field	Membrane	Description
Model construction		✓	✓					Loop modification or molecular assembly
Mechanical deformation		✓	✓					Elastic network deformation (e.g., AFM simulation)
Ion binding sites	✓		✓			✓		Ion surface scanning with electrostatics
Small ligand binding sites	✓	✓	✓		(✓)	✓		Pocket detection at the surface of a biomolecular target
Docking	✓	✓	✓		(✓)	(✓)		Flexible docking with Coulomb/Poisson‐Boltzmann electrostatics
Membrane insertion		✓		✓	✓		✓	Rigid body or elastic model in implicit membrane
Multimeric assembly				✓	✓		✓	SASA‐driven assembly of rigid body monomers in implicit membranes
Volume fitting		✓	✓			✓		Elastic model positioning in volume or density map

*Note*: Check marks (✓) indicate active features from Table 1. When a checkmark is in parentheses, it denotes optional features. Table under CC‐BY license by Marc Baaden.

**FIGURE 3:**
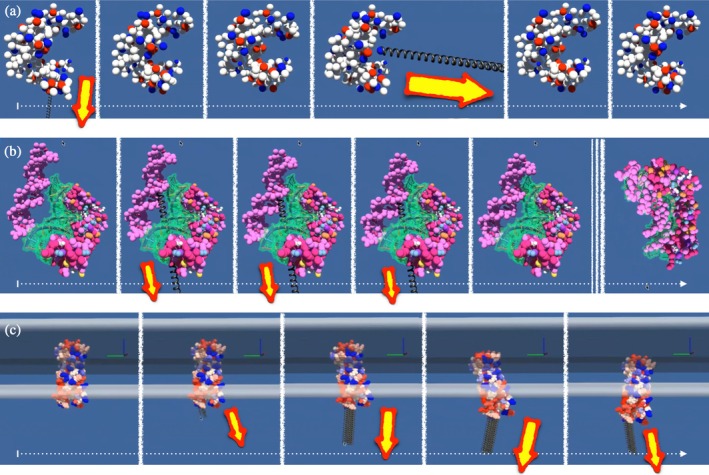
Time‐lapse sequences of three interactive experiments. The user's manipulations are represented by gray springs. The size of the spring reflects the amplitude of the force. To further highlight all interactions, yellow arrows with a red border can be seen on the panels, pointing in the direction in which a force is exerted, with their size being proportional to the force. The progression over time is indicated by a white horizontal arrow. The different points in time within a panel are separated by white vertical lines. (a) Investigation of the mechanical properties of guanylate kinase. First the user pulls downwards, then allows the system to relax before pulling harder to the right and observing the relaxation again. The beads are colored according to their charge (blue positive, red negative), with the radii indicating size exclusion. (b) Docking of a flexible pink DNA fragment to a rigid protein. In this test system used for training, the crystal structure of the complex, that is, the solution of the docking experiment, is known and is highlighted by a transparent green surface to guide the user. After the user has applied force to bring the DNA closer to the protein in three frames, the system relaxes towards the solution, driven by an electrostatic field, without applying any further force. (c) Repositioning of the OmpA membrane protein in an implicit lipid bilayer indicated by two horizontal gray planes in the upper part of the panel. The user starts to pull the protein out of the membrane by overcoming the membrane insertion forces. The Image is under CC‐BY License by Marc Baaden.

Three application types from Table 2 are now presented in more detail, and screenshots of representative interactive manipulations that support such applications are shown in Figure 3.

### 3.2 Investigation of the conformational properties of guanylate kinase

To demonstrate the interactive capabilities of the BioSpring framework, we first investigate the enzyme guanylate kinase (GK) (Agarwal et al. 1978; Oeschger 1978). This essential enzyme transfers a phosphoryl group from ATP to guanosine monophosphate, creating guanosine diphosphate. This reaction is crucial for nucleotide balance in cells and plays a key role in RNA/DNA synthesis. Together with the closely related adenylate kinase, GK serves as a model system for the study of protein dynamics and the time scales of conformational changes in enzymes. Their simple structure, well‐defined catalytic mechanisms, and dynamic behavior make them ideal for studying the transition between functional states, which is fundamental for understanding enzyme catalysis, allostery, and molecular recognition (Henzler‐Wildman et al. 2007a).

GK is characterized by a characteristic U‐shaped conformation. The model combines elastic network constraints with non‐bonded interactions, allowing for realistic biomolecular behavior while maintaining computational efficiency. It corresponds to the mechanical deformation example of Table 2. We constructed the system with a coarse‐grained representation where amino acid residues are represented as beads connected by elastic springs. In addition to the traditional elastic network connections, the model accounts for both steric and electrostatic interactions between the beads, with charged residues highlighted in the visualization. This hybrid approach captures both large‐scale conformational dynamics and local physicochemical interactions. Real‐time manipulation of such a protein model as depicted in Figure 3a reveals several important features. Long‐range mechanical coupling is observed when force is applied to specific regions of the protein, while perturbations propagate across the structure through the elastic network, revealing long‐distance allosteric effects (Choi and Zocchi 2007; Tseng et al. 2010) typical of protein dynamics. The simulation successfully reproduces the biological opening and closing motion of GK (Delalande et al. 2011; Henzler‐Wildman et al. 2007b; Sacquin‐Mora et al. 2010), which is essential for its enzymatic function. The model exhibits inherent structural elasticity—when deformed within biological limits, it returns to its equilibrium conformation, which is controlled by both the elastic network and non‐bonded interactions. The interactive manipulation exhibits complex dynamic behavior and responsiveness. Under certain deformations, the system can adopt metastable configurations, especially when residues are pulled through other parts of the structure. These states persist due to non‐bonded interactions that generate local energy minima, although the system's own conformational memory favors the reference state. If the user provides sufficient activation energy, the system can overcome structural perturbations on its own through the combined action of elastic restoring forces and non‐bonded interactions and eventually return to its equilibrium state. Such an interactive simulation provides valuable insights into protein mechanics and enables intuitive assessment of structure–function relationships through real‐time reactions and visual feedback.

### 3.3 Elastic protein–DNA docking

Another compelling application is interactive protein–DNA docking with elastic network models. It serves as an exemplary system for deciphering macromolecular recognition mechanisms. Despite their fundamental biological importance, these complexes are still largely unresolved. The challenge lies in critical conformational changes, where DNA often requires a specific deformation to achieve binding, coupled with the subtle interplay between structural complementarity and electrostatic interactions. This use case shown in Figure 3b demonstrates the integration of elastic networks with additional tools to facilitate molecular assembly processes. The system consists of a protein target and a DNA fragment, where the binding site is visualized by a green surface indicator that marks the desired interaction region. In this validation experiment, the interaction region was previously identified qualitatively and to some extent quantitatively through experimental or theoretical methods (single mutation, cross‐link, NMR‐STD, evolutionary conserved amino acids…). It is one of the protein–DNA complexes from the representative set described in Poulain et al. (2008). The simulation uses elastic networks for both the protein and DNA components to maintain their structural integrity while allowing necessary conformational adjustments during the docking process. A notable feature of this implementation is the inclusion of a guiding field that supports the docking process, a variation of the docking example of Table 2. This field introduces additional forces that help guide the DNA fragment to its correct binding position on the protein surface. The interactive docking process combines user‐guided manipulations with automatic refinement. During initial positioning, users can interactively manipulate the DNA fragment to bring it close to the target protein's binding site. This manipulation requires careful control, as excessive forces can destabilize the simulation. The refinement of the assembly is controlled by a pre‐calculated potential field. Once the DNA fragment is positioned close to the binding site, this guiding field becomes increasingly influential. At this point, the system shows an autonomous ability to refine the positioning, with the DNA fragment naturally orienting itself towards the optimal binding configuration: the experiment culminates in spontaneous assembly of the final complex, without further user intervention. The implementation reveals several important technical aspects regarding force sensitivity, delayed start, and dimensional constraints. The fact that this simulation requires careful force management during manipulation to maintain stability underscores the delicate balance required in interactive molecular modeling. The system employs a 10‐s loading timer to ensure proper initialization of the guide field before enabling visualization and interaction. While the current experiment was conducted in a 2D interface, a virtual reality implementation could provide more intuitive control for such complex 3D manipulations. This molecular docking implementation demonstrates the potential for investigating and visualizing molecular assembly processes. The combination of user‐guided positioning and physics‐based refinement offers an intuitive yet physically sound method for probing biomolecular interactions.

### 3.4 Insertion of proteins into implicit membrane models

The insertion of membrane proteins into lipid bilayers represents a major computational challenge due to the complex interplay between protein structure and membrane environment. This process has been extensively discussed and documented in the literature, but studying the rugged energy landscape of this process in an interactive way and in real time is an unprecedented approach. We illustrate the framework's capabilities in addressing these challenges through an interactive simulation of OmpA, a well‐characterized outer membrane protein (Bond et al. 2006; Cox et al. 2008). By combining implicit membrane representation with rigid body dynamics (Table 2), our system enables real‐time investigation of protein–membrane interactions while maintaining computational efficiency. The simulation environment provides immediate visual feedback as captured in Figure 3c. The visualization can be enhanced through both structural visualization and quantitative analysis of key parameters via an optional live plotting interface. The simulation environment allows direct manipulation of the orientation and insertion depth of the membrane protein. The implementation includes adjustable force parameters to fine‐tune the interaction strength between the protein and the implicit membrane. These customization options allow the user to avoid overshooting during manipulation while maintaining sufficient forces for realistic protein–membrane interactions. The system reveals several key features of membrane protein behavior. When the protein deviates from its preferred orientation, it returns to a characteristic tilted configuration in the membrane. Attempts to maintain the protein in alternative orientations, such as a fully vertical orientation or a horizontal position within the membrane plane, result in the system returning to its preferred tilted state. This behavior indicates the presence of well‐defined energetic preferences for specific insertion angles. Real‐time analysis is facilitated by the interactive display of key parameters such as insertion depth and orientation angle. This feature provides immediate feedback on the protein's behavior and allows systematic studies of the insertion parameters. The visualization includes multiple views, especially side perspectives, which are critical for monitoring the protein's tilt angle relative to the membrane normal. The calibration of the forces proves to be crucial to achieve realistic behavior while maintaining the stability of the simulation. The system demonstrates both the capability for local conformational sampling and the ability to recover from significant perturbations, such as partial extraction from the membrane. While the depicted experiment utilizes a planar implicit membrane, the framework is extensible to arbitrary membrane geometries and thus offers potential for the investigation of more complex biological scenarios. The approach is efficient for studying preferred orientations and insertion depths.

## 4 DISCUSSION

The interactive and lightweight approach to molecular modeling enabled by the BioSpring framework offers several distinct advantages, yet has some limitations that should be discussed. The main strength of the framework lies in its ability to combine the user's expertise with real‐time feedback, allowing intuitive exploration of complex molecular systems. The interactive nature of the approach provides immediate visual and quantitative feedback that allows the user to develop an intuitive understanding of the behavior of molecular systems. This is particularly evident in the insertion of membrane proteins, where the user can directly observe and influence protein orientation preferences. In protein–DNA docking scenarios, the combination of user guidance and physics‐based refinement enables efficient sampling of possible binding configurations.

The versatility of the framework is evident when applied to complex integrative modeling workflows. Two challenging case studies—the ryanodine receptor (RyR) (Molza et al. 2022) and the dystrophin filament (Delalande et al. 2018)—demonstrate our ability to handle large, structurally diverse macromolecules. In the case of the RyR, interactive fitting guided by experimental density maps enabled the system to overcome fundamental modeling challenges, including structural gaps and dynamic interface regions. This integration of experimental and theoretical approaches enabled the development of coherent models for different functional states. The study of the filamentous architecture of dystrophin, particularly in the context of Becker muscular dystrophy‐associated deletions, demonstrated the ability of the BioSpring simulation engine to combine SAXS data with interactive modeling. These examples underscore the effectiveness of the BioSpring framework in combining diverse quantitative data (often more straightforward to obtain from computational and simulation approaches) and heterogeneous qualitative data from various experimental techniques. Combining these data types is usually a challenge; however, the BioSpring framework facilitates this integration so that complex biological questions can be answered. In addition, other types of data, for example, from NMR experiments or FRET measurements, can be seamlessly integrated into such studies.

The framework's interactive capabilities extend to immersive virtual reality (VR) environments, where its real‐time responsiveness enables intuitive manipulation of human‐scale molecular assemblies (Baaden 2022). This immersive approach particularly benefits the modeling of complex systems such as membrane proteins and large ion channels, where spatial understanding is crucial.

The interactive approach accelerates the initial handling of molecular systems. It plays a complementary role to traditional computational methods. This preparatory phase, which incorporates human expertise and intuition, helps to guide subsequent detailed simulations through informed decisions. For example, when creating initial models, the BioSpring framework facilitates rapid structural changes such as the repositioning of loops or the assembly of molecular fragments. When studying molecular deformation, the interactive probing of rigid and flexible regions can provide important insights for planning advanced simulations. While one can quickly identify potential conformational states or binding sites, their detailed characterization usually requires additional validation by methods such as molecular dynamics simulations. By integrating such interactive steps early in the modeling workflow, researchers can optimize their computational resources and make more informed decisions about which detailed calculations to perform, effectively combining human insight with computational power. A notable limitation of the interactive approach is the inherent user dependency of the discovery process and the lack of reproducibility. Different users may probe the same system differently, and the quality of the results may depend on the user's experience and attention. However, this limitation is offset by the framework's ability to utilize human insight and expertise in guiding the process.

To ensure the widespread adoption and use of the BioSpring simulation engine, we have prioritized accessibility by adhering to principles for sharing methods and algorithms that we previously designed. By providing binaries, Docker images, source code, and most importantly, the turnkey MolPlay interface, we aim to enable researchers from different technical backgrounds to easily test, customize, and apply our software to their specific research needs. This multi‐layered approach removes significant technical hurdles.

Looking to the future, the BioSpring framework promises a range of new applications. Due to its ability to process multiscale representations, it is particularly suitable for studying large molecular assemblies such as membrane protein complexes or nucleoprotein structures. Future developments could focus on improving quantitative analysis capabilities and implementing more sophisticated visualization techniques, such as volume rendering, especially for complex multi‐component systems. To further extend its molecular modeling flexibility, the framework could implement support for more refined side‐chain behavior and their rotameric states (Jacobs et al. 2001), but also dihedral angles on the backbone, offering insights into conformational variability and enhancing dynamic analysis. To address the above‐mentioned limitation of user dependency, systematic session recordings and analysis functions are planned. These recordings could be complemented by training AI agents through imitation learning, enabling automated exploration of conformational spaces while preserving the intuitive strategies developed by human experts during interactive molecular simulations, as introduced by Glowacki's group (Dhouioui et al. 2024). These additions will allow users to document, share, and reproduce their interactive manipulation paths to promote reproducibility across different sessions and users.

## 5 CONCLUSION

BioSpring introduces a versatile framework for interactive molecular modeling that bridges the gap between structure visualization and physics‐based simulation. With its modular architecture and multi‐scale visualization capability, the framework enables real‐time testing of diverse molecular systems while maintaining physical accuracy within the limits of interactive performance. The three demonstrated applications—protein conformational dynamics, protein DNA docking, and membrane protein insertion—illustrate the broad utility of the framework in different areas of structural biology to study complex molecular behaviors. The architecture of BioSpring's simulation engine is designed to be extensible, allowing the integration of additional physical models and interaction schemes as computational capabilities increase. As interactive molecular modeling evolves in parallel with improvements in computational resources, such interactive frameworks will play an increasingly important role in bridging the gap between human understanding and phenomena at the molecular level.

## AUTHOR CONTRIBUTIONS

**Benoist Laurent:** Investigation; software; data curation; writing – review and editing; methodology. **André Lanrezac:** Investigation; software; data curation; methodology; visualization; writing – review and editing. **Hubert Santuz:** Resources; software; data curation; writing – review and editing. **Nicolas Ferey:** Software; investigation; methodology; writing – review and editing. **Olivier Delalande:** Investigation; software; data curation; methodology; visualization; writing – review and editing. **Marc Baaden:** Conceptualization; investigation; writing – original draft; writing – review and editing; methodology; validation; visualization; software; formal analysis; funding acquisition; supervision; project administration.

## CONFLICT OF INTEREST STATEMENT

The authors declare no conflicts of interest.

## Data Availability

We as authors state that all figures and data tables in this article are our own (i.e., Tables 1 and 2 and Figures 1, 2, 3) and remain under our own personal copyright, with the permission to be used here. We also make them available under the Creative Commons Attribution 4.0 International (CC BY 4.0) license and share them at https://osf.io/5mq87/. The data that support the findings of this study and led to the generation of the figures and tables are openly available on the BioSpring and MolPlay websites (https://github.com/LBT-CNRS/biospring).
